# Designing of guava quality classification model based on ANOVA and machine learning

**DOI:** 10.1038/s41598-025-09684-7

**Published:** 2025-09-30

**Authors:** Abiban Kumari, Jaswinder Singh

**Affiliations:** Department of Computer Science and Engineering, Guru Jambheshwar University of Science and Technology, Hisar, Haryana 125001 India

**Keywords:** Machine learning, SVM/KNN/ANN/RF, Food quality, Segregation, Guava maturity classification, ANOVA, Machine learning, Quality control

## Abstract

The precise maturity quality classification of guava is crucial at farm level, retail, storage, and supply chain. The manual classification causes substantial postharvest losses, which increases the demand for material and resources. Therefore, the present study proposed an automated, precise, and accurate model for quality classification according to the maturity stages (Green, Mature Green, Ripe) of three varieties (Local Sindhi, Riyali, Thadhrami) of guava. The study aimed to develop a precise, accurate and automated model for the classification of guava according to their maturity stages. The guava images were used to extract color, shape, and texture features. Analysis of Variance (ANOVA) was used for the selection of important features. The six different machine learning (ML) classifiers; Artificial Neural Network (ANN), k-Nearest Neighbor (KNN), Support Vector Machine (SVM), Cubic SVM, Quadratic SVM, and Random Forest (RF) were used to find out the best classifier for maturity classification. Among the proposed classifiers, the RF classifier was found to be the best classifier for all three varieties of guava. The Quadratic SVM classifier showed the lowest classification accuracy. The study concluded that RF classifier was found to be a robust model for the maturity classification of guava.

**Supplementary Information:**

The online version contains supplementary material available at 10.1038/s41598-025-09684-7.

## Introduction

Guava is a nutritionally rich and commercially viable fruit crop, is being grown in tropical and subtropical regions around the globe. Globally, India is the largest producer of the Guava, contributing to approximately 44% of global production^1^. Guava is a source of important nutrients such as vitamin C, Vitamin B, Vitamin E, dietary fiber, minerals and phenolic compounds. The perishable nature of the guava resulted in high postharvest losses up to 30%, during marketing, storage and transportation^1–3^. Due to climacteric in nature the quality of the fruit changes continuously with the maturity stage, which affect the fruit storage, transportation, and consumer acceptability. Thus, the grading of the guava based on their maturity or ripeness level is very important to increase the profitability and marketability in the local and export market^4^. The maturity stage affect texture, sweetness, shelf life and disease susceptibility due to metabolic activity in fruit thus affecting the postharvest quality of the guava. So far, the maturity grading of the guava has been mostly carried out manually by the labor. The manual grading substantially increases the demand of human and material resources^5^. Therefore, an automatic, specific and precise grading system must be solemnly taken into consideration to mechanize the maturity and ripeness classification of guava to ensure the efficient postharvest management and marketing. The ripening stage of the fruit can be identified by two different approaches that is destructive and non-destructive methods. The non-destructive methods allow the accurate and rapid quality evaluation of fruit based on internal and external quality attributes. The destructive method consists of determination of the internal quality attributes such as fruit firmness, total soluble solids, titratable acidity, acid and sugar etc. In contrast, the non-destructive methods such as RGB imaging, hyperspectral and multispectral imaging are much more efficient and economical, enabling the fruit ripeness identification without the destruction of fruit^6,7^. Therefore, researchers are continuously trying to develop a non-destructive, simple, rapid, assessable and accurate method to classify the maturity and ripeness of fruits. Recently, the deep learning technique has also been showed wide applications in the horticulture sector such automatic grading and sorting, defect and disease identification and shelf life prediction by acquiring fruit images^8^. Generally, in these types of tasks, different types of models are trained from the images of the single fruit to predict the fruit ripeness. The convolution neural network (CNN) was used to differentiate the ripeness stages of banana and compared with CNN using transfer learning. Beside this, number of studies were employed to predict the different stages of fruit (mulberries^9^tomato^10^ and grapes^11^) ripeness using DenseNet, AlexNet, and VGG respectively. Further, few studies showed the deep learning-based method for the classification of fruit ripeness using multiple fruits in a single image. The external properties such as color, shape, size and surface texture were used in the optical imaging method. The previous studies showed that machine learning techniques have been used for the identification of guava, variety classification of guava, classification of disease and healthy guava^4,12–14^.

The previous research lacks on an important aspect of postharvest quality classification of the guava based on its maturity stages. Therefore, the novelty of the present study is the development of a precise and accurate model for determining the maturity stages of guava to evaluate the postharvest quality, improve fruit marketability and consumer acceptability, and reduce postharvest losses. Further, the selection of the features was optimized to eliminate the information redundant and improve the effectiveness and efficiency of the classifiers, which is inadequately addressed in the previous study.

Therefore, the present study aimed to develop a precise and accurate model for the quality classification of maturity stages of three different varieties of Guava. The classification was performed using six different classification models (RF, KNN, ANN, SVM, cubic SVM, and quadratic SVM) to find out the most accurate classifier. To enhance the performance of the model, one-way ANOVA was used for the selection of features.

## Materials and methods

### Data

**Fig. 1:**
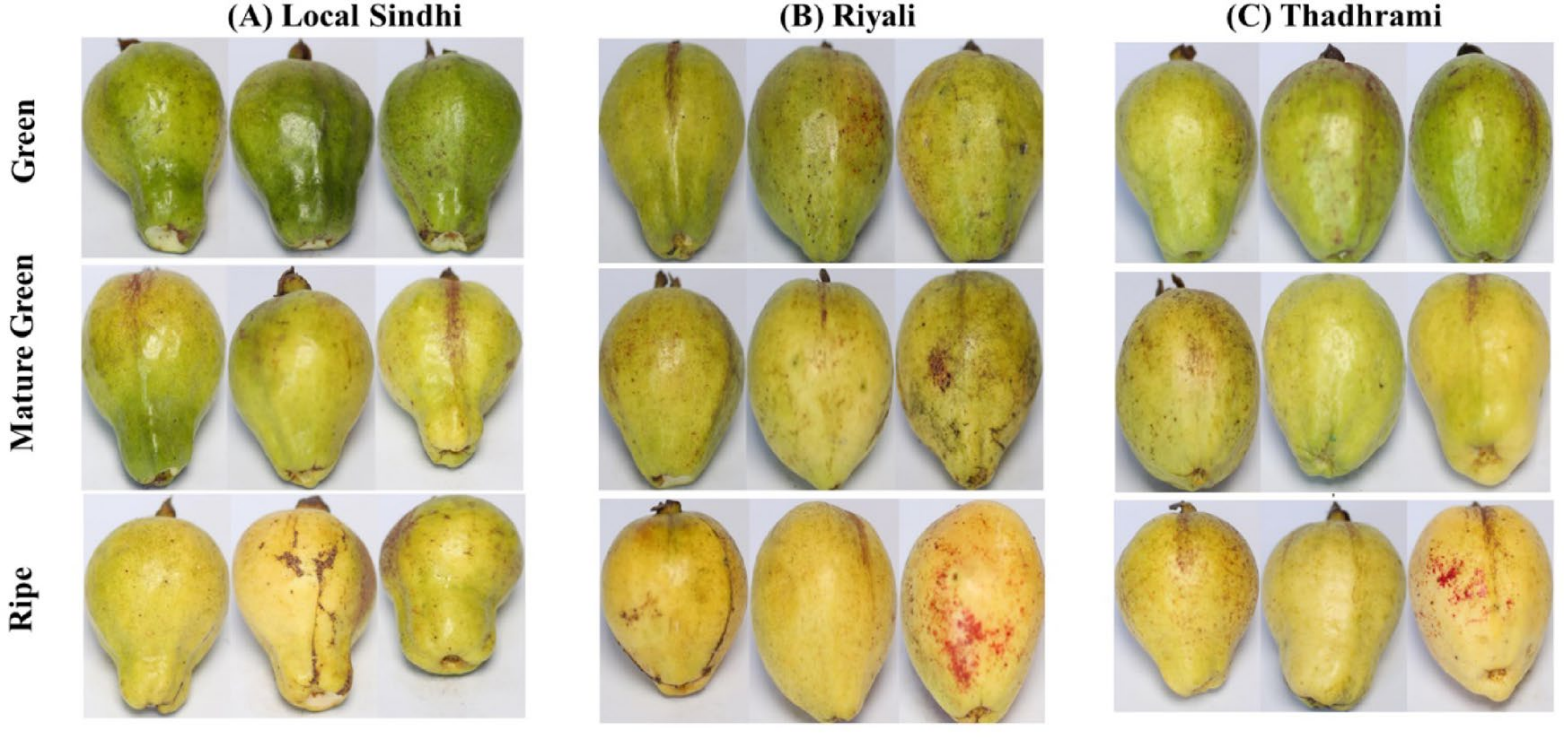
Guava images of the different varieties (**A**) local Sindhi (**B**) Riyali (**C**) Thadhrami at different maturity stage (green, mature green and ripe).

**Fig. 2:**
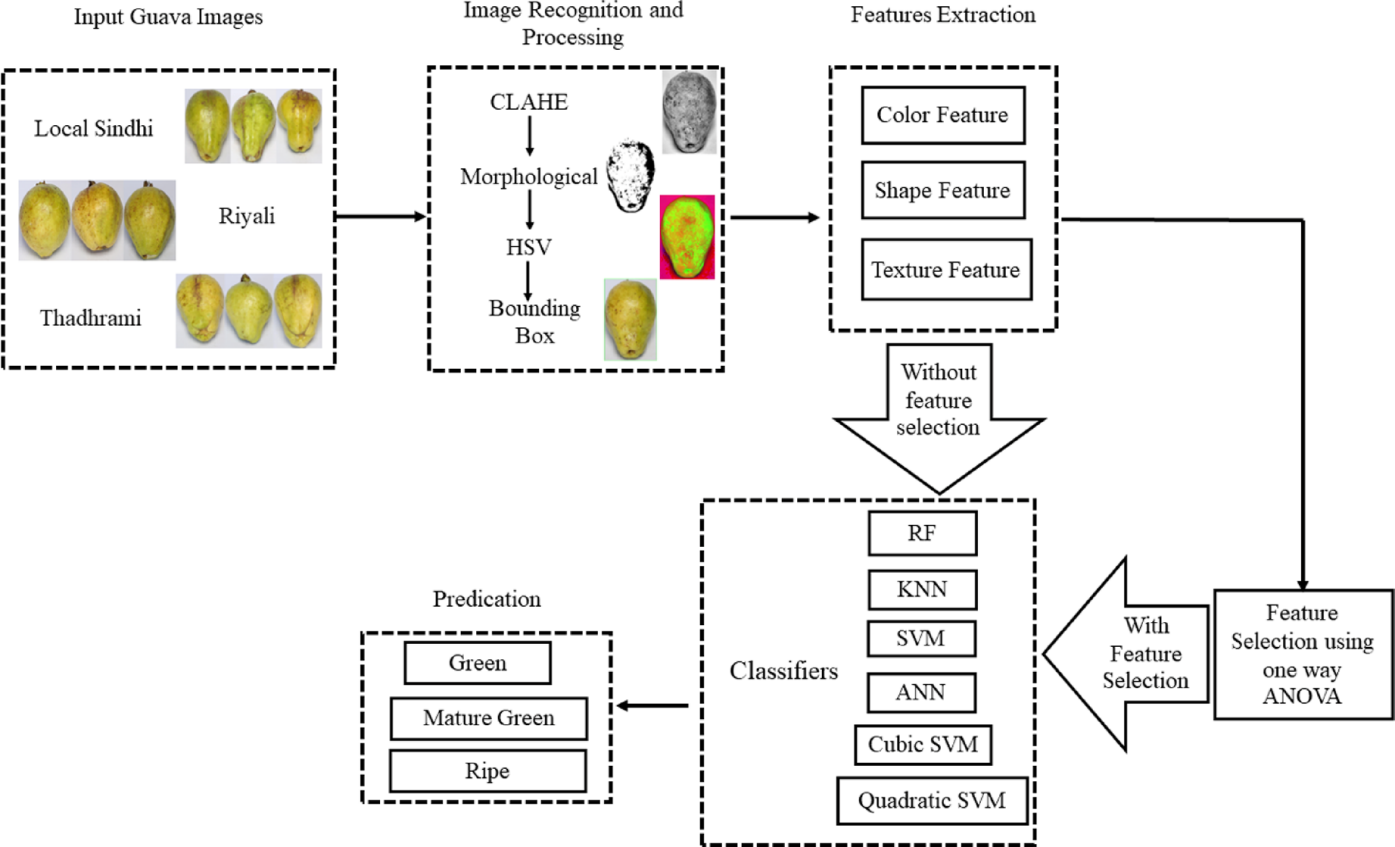
The proposed methodology flow diagram for maturity classification of guava.

The dataset used in the present study was the images of three different varieties (Local Sindhi, Riyali, and Thadhrami) of guava^15^. The images dataset was classified into three different categories according to their maturity level (a) Green guava, (b) Mature green guava and, (c) Ripe guava in each variety^15^ (Fig. 1). The dataset consists of total 2309 images of the guava in the jpg format^15^. The detailed description about the dataset is provided in the supplementary Sect. 1. This dataset was used for the designing of a precise and accurate machine learning model for the quality classification of guava based on their maturity levels (Fig. 2).

### Image processing

The pre-processing of data was performed to extract the desired feature. In the present study, the openly available image dataset from the data in brief was used for the maturity classification of the guava fruits^15^. A canny edge detector was used to determine the fruit edge. The morphological operations were extracted, and the region of interest (ROI) was performed.

**Fig. 3:**
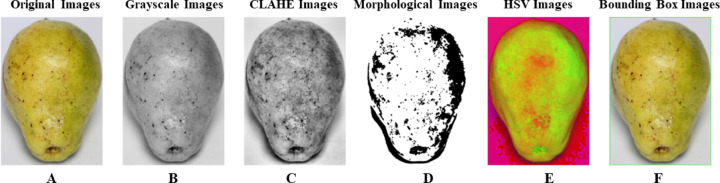
Image preprocessing process (**A**) original image (**B**) grey level image (**C**) CLAHE image (**D**) morphological processing (**E**) HSV transform (**F**) bounding box (drawing contours).

The noise from the background was eliminated by using the R, G and B channels^15^. The image representation and enhancement were performed by using Contrast Limited Adaptive Enhancement (CLAHE) techniques^16^. The Hue, Saturation, Value (HSV) color spaces were used to enhance the color thresholding (Fig. 3). Further, the component V was used to improve the brightness information, and H and S component thresholding were assigned for further isolation. The morphological operation of open, close, fill and dilation were performed to eliminate the remaining noise^17^. To determine the region of Interest, the bounding box techniques was used to identify the object^18^.

The analysis was performed by using a 2.5 GHz Intel Core i5- 12,500 H CPU, HP Laptop equipped with 16 GB RAM, NVIDIA GEFORCE RTX and Python. The image analysis involves extracting features, selecting them through one-way ANOVA, and implementing different classifiers for classification to predict the maturity classes of fruits.

### Feature extraction

The objective of feature extraction was to convert data into a representative form for further classification task. The color^19^shape^20^ and texture^21^ features from guava images were identified as major feature vectors and these features were extracted from the guava images. The color represents the outer appearance of the guava while shape and texture represent the geometric configuration of the guava. Therefore, the color features were extracted using segmentation, while the shape and texture were extracted using geometrical characteristics of the images. Further the analysis was performed to differentiate the fruits and background noise.

#### Color features

The color histogram is used to extract the color features by using the number of pixels in the selected color space like RGB or HSV. The RGB features were further used to extract the HSV features. The main HSV color features were Hue (H), Saturation (S), and Value (V)^19^. The RGB color space was utilized to create a wide spectrum of colors with channels 0–255. HSV color spaces were then applied to RGB images^22^. The transfer of the RGB to HSV color spaces was performed as provided in the Eqs. (1), (2) and (3).

1 $$\:V=\text{M}\text{a}\text{x}(R,\:G,\:B)$$

2 $$H = ~\left\{ {\begin{array}{*{20}l} {undefined~,} \hfill & {if~Max = Min} \hfill \\ {\left( {\frac{{G - B}}{{Max - Min}}} \right)~X~A,~} \hfill & {~if~Max = R} \hfill \\ {\left( {\frac{{B - R}}{{Max - Min}} + 2} \right)~X~A,} \hfill & {if~Max = G} \hfill \\ {\left( {\frac{{R - G}}{{Max - Min}} + 4} \right)~X~A,~} \hfill & {if~Max = B} \hfill \\ \end{array} } \right.$$

3 $$S = ~\left\{ {\begin{array}{*{20}l} {0,} \hfill & {~if~Max = Min} \hfill \\ {Max - Min~,~} \hfill & {others} \hfill \\ \end{array} } \right.$$

where, the RGB color space the values of R, G and B varied from 0 to 255.

If H is given in radians, then A = π/3; if expressed in degrees, then A = 60°. Min = min (R, G, B), Max = max (R, G, B). In radians, the range of H is − π/3 to + 5π/3, while in degrees, it ranges from − 60° to 300°. The S and V, however, range from 0 to 255.

#### Shape features

The shape of the fruit is an important feature. The main component to be extracted for the shape features were area of the fruit, centroid x, centroid y, perimeter, axis length (minor and major), orientation, solidity, and eccentricit^21^. The technique involves converting the image to grayscale, followed by binary thresholding to isolate the object of interest. Contours were detected to extract the object’s border (to join the boundary points of an image) and essential shape properties such as area, centroid, axis lengths, orientation, perimeter, solidity, and eccentricity. The selection of these traits was driven by their capacity to characterize the object’s shape comprehensively.

#### Texture features

The process of texture features extraction evaluates the GLCM^23^ and LBP^24^ features application to compose the contrast, homogeneity, energy, variance, smoothness, root mean square (RMS), correlation, kurtosis, skewness, entropy, mean and standard deviation of LBP and GLCM^25^. LBP texture features evaluate the labels of the image pixels by performing thresholding of each pixel’s neighborhood. LBP operate the local textural features of an image^26^. The LBP describe the difference between the neighboring pixel ($\:{n}_{i}$) and center pixel $\:\left(c\right)$ on the guava fruit images.

where $\:P$ represent number of neighborhood pixels, $\:{n}_{i}$ denote neighboring pixel, and $\:{G}_{c}$ denote the center pixel, and N, consist of total number of neighbors and R is the neighborhood radius^26^. The size of histogram features is $\:{2}^{P}$ that is extracted from given LBP code in Eqs. 4 and 5.

4 $$LBP_{{N,R~}} = ~\mathop \sum \limits_{{i = 0}}^{{P - 1}} s\left( {n_{i} - G_{c} } \right)2^{P}$$

5 $$s\left( x \right) = \left\{ {\begin{array}{*{20}l} {1,~} \hfill & {if~x> 0} \hfill \\ {0,~} \hfill & {~othervise~} \hfill \\ \end{array} } \right.$$

The binary labels (0,1) of neighbor pixels depended on whether the center pixel had a higher intensity than the adjacent pixel. A statistical approach GLCM was used to extract the texture feature from images and considers the spatial relationships between the pair of image pixels^26^.

A matrix characterizing the presence of pixels next to each other (from the right) in a greyscale image was created using GLCM features, as given below;

6 $$Contrast = ~\mathop \sum \limits_{{i,j}} \left( {i - j} \right)^{2} P\left( {i,j} \right)$$

7 $$Correlation = ~\frac{{\mathop \sum \nolimits_{{i,j}} \left( {i - \mu _{i} } \right)\left( {j - \mu _{j} } \right)P\left( {i,j} \right)}}{{\sigma _{i} \sigma _{j} }}$$

8 $$Energy = ~\mathop \sum \limits_{{i,j}} P\left( {i,j} \right)^{2}$$

9 $$Homogeneity = ~\mathop \sum \limits_{{i,j}} \frac{{P\left( {i,j} \right)}}{{1 + \left| {i - j} \right|}}$$

10 $$Entropy = ~\mathop \sum \limits_{{i,j}} p_{i} {\text{log}}\left( {p_{i} } \right)$$

11 $$Variance = ~\frac{1}{N}\mathop \sum \limits_{{i = 1}}^{N} \left( {x_{i} - \mu } \right)^{2}$$

12 $$Kurtosis = ~\frac{1}{N}\mathop \sum \limits_{{i = 1}}^{N} \left( {\frac{{x_{i} - \mu }}{\sigma }} \right)^{4} - 3$$

13 $$Skewness = ~\frac{1}{N}\mathop \sum \limits_{{i = 1}}^{N} \left( {\frac{{x_{i} - \mu }}{\sigma }} \right)^{3}$$

GLCM consist of i, x, j, x, and n matrix. Where $\:P(i,j)$ GLCM elements at position (i, j) and n be the total number of GLCM elements. Following the extraction of the GLCM matrix, the following features were computed: energy, homogeneity, variance, skewness, kurtosis, contrast, correlation, and entropy^27^. Where $\:{\mu\:}_{i},\:{\mu\:}_{j}\:$was represent the mean and $\:{\sigma\:}_{i},{\sigma\:}_{j}$ represent the standard deviation of $\:P\left(i,j\right).\:$ $\:{x}_{i}\:$ be the pixels intensities and N be the number of pixels. This integrated feature set enables the classifier to use both geometric and textural information, leading to enhanced accuracy in object classification tasks.

### Feature selection using ANOVA

The maturity stage classification was performed in two different ways; without feature selection and feature selection using one-way ANOVA, to determine the variance between different groups. The multi-comparison one-way analysis was used for the selection of features with *P*-value < 0.05^13^. The mean separation was subsequently established using Tukey’s honestly significant difference test, which showed that one or more features were significantly different. The selection of the features decreases the data dimensionality by choosing the subset of the measured features to develop a machine learning model to obtain the optimized classification performance.

### Fruit classification

In the present study, six different ML classifiers (Random Forest, KNN, SVM, Cubic SVM, Quadratic SVM, ANN) were used to select the best model for the maturity classification of guava, as discussed below;

#### Artificial neural network

A feedforward backpropagation technique was applied during the ANN network’s training phase to remove the background noise^28^. In ANN network gradient descent was used to minimizes the error by altering the weights linking the neurons. Each layer of the MLP for an ANN was made up of a set number of predetermined neurons, with 29 input neurons, 16 hidden neurons, and 3 output neurons.

The input layer provides the data to the hidden layers neuron, *j*, and sums the input signals xi, after weighting them with the strength of their corresponding connections $\:{w}_{i,j}$ in the input layer. Then the obtained output was further forwarded to the output layer, $\:{y}_{i}$, as a function f of the sum as presented in the Eq. (14);

14 $$\:{y}_{i}=f\:\left(\sum\:{w}_{i,j}{x}_{i}\right)$$

#### **k-Nearest neighbours** (KNN)

The KNN method operates on the premise that the instances belonging to each class were mostly surrounded by same class instances. Thus, it was used to provide with a collection of training samples in the feature space and a scalar value k^29^. The classification of an unlabeled instance was determined by label assigning that appears closest to that instance generally in the k training samples. Among the metrics used to quantify the distance between instances, the Euclidean distance was the most extensively adopted.

The Euclidean distance was determined using the equation given below;

15 $$L\left( {x_{i} ,~x_{j} } \right) = \left( {\mathop \sum \limits_{{i,j = 1}}^{n} \left( {\left| {x_{i} - ~x_{j} } \right|} \right)^{2} } \right)^{2} \;{\text{where}},\;~x \in ~R^{n}$$

$\:{R}^{n}$ represented the n tuples of real numbers, $\:{x}_{i},\:{x}_{j}$ are n-dimensional points with n coordinates.

#### Random forest

The RF is an efficient method for classification and regression that may effectively categories a huge dataset. The decision trees are built in an ensemble by this algorithm. Grouping weak learners to develop a strong learner is the primary idea of ensemble strategies. As the input was entered at the top and progresses down the tree, the original data was randomly sampled and substituted into progressively smaller groups. Random forest trees with an arbitrary number were utilized to calculate the sample class. The forecasts of the RFs were believed to be the majority votes of all of the trees’ projections for classification^29^.

The feature space was divided into M regions, Rm, where 1 ≤ m < M, by a decision tree with M leaves. The prediction function f(x) for every tree is defined as follows:

16 $$f\left( x \right) = ~\mathop \sum \limits_{{m = 1}}^{M} c_{{m~}} ~\Pi \left( {x,~R_{m} } \right)$$

Let M be the number of regions in the feature space, $\:{R}_{m}$ denote a region of adequate size for m, and cm be a constant that is suitable for m.

17 $$\Pi \left( {x,~R_{m} } \right) = ~\left\{ {\begin{array}{*{20}c} {1,\quad if~x \in R_{m} ~} \\ {0,\quad otherwise} \\ \end{array} } \right.$$

A final classification was determined by aggregating the votes of the majority of all trees.

#### Support vector machine

The SVM was used for the collection of data and to determines the largest margin in the high-dimensional feature space by utilizing the principle of construction risk minimization using quadratic, cubic, and radial basis kernel functions^30^. The kernel function influences the performance of classifier such as efficiency and accuracy of the classifiers. The training and testing data, made up of several data instances with one target value and different attributes, were used to build the model. The label reorganization specifies the model correctness by providing an indicator of good outcome, thus validate the algorithm accuracy.

The SVM goal was to optimize the margin by locating the optimal separation hyper plane; as shown in Eqs. 18 and 19.

18 $$\:f\left(x\right)=\:{w}^{T}x+b$$

Where, $\:{w}^{T}x=\:{\sum\:}_{i}{w}_{i}{x}_{i}$. The vector w is referred to as the weight vector, and the vector b is referred to as the bias vector. x represents a vector with components $\:{x}_{i}$. The notation $\:{x}_{i}$ will signify the ith vector in a dataset consisting of n labelled.

19 $$\:k\left(x,\:{x}^{{\prime\:}}\right)={({x}^{T}{x}^{{\prime\:}}+1)}^{d}$$

For this kernel, all monomials with degree less than or equal to d make up the feature space. The kernel that has a value of d = 1 for linear, d = 2 denotes quadratic and d = 3 for cubic kernels^30^.

#### Model performance evaluation

The model performance was evaluated by comparing the prediction results of the proposed model with the true labels. In the present study, the accuracy, precision, recall, F1-score and specificity were used as quantitative indicators for model performance, Eqs. 20–24. The different terms are defined as follows: False Positive (FP) is an incorrect classification of negative cases, and False Negative (FN) is an incorrect classification of the positive cases. True Positive (TP) was the correctly classified positive cases of true fruits classification and True Negative (TN) was the correctly classified negative cases of incorrect fruit classification. The higher values of precision and recall lead to less values of false positives and false negatives.

20 $$\:\text{A}\text{c}\text{c}\text{u}\text{r}\text{a}\text{c}\text{y}=\frac{TP+TN}{TP+FP+TN+FN}$$

21 $$\:\text{S}\text{p}\text{e}\text{c}\text{i}\text{f}\text{i}\text{c}\text{i}\text{t}\text{y}\:=\frac{TN}{TN+FN}$$

22 $$\:\text{P}\text{r}\text{e}\text{c}\text{i}\text{s}\text{i}\text{o}\text{n}\:=\frac{TP}{TP+FP}$$

23 $$\:\text{R}\text{e}\text{c}\text{a}\text{l}\text{l}\:=\frac{TP}{TP+FN}$$

24 $$\:\text{F}1\:\text{s}\text{c}\text{o}\text{r}\text{e}\:=\frac{2*\text{P}\text{r}\text{e}\text{c}\text{i}\text{s}\text{i}\text{o}\text{n}*Recall}{Precision+Recall}$$

## Results and discussion

**Fig. 4:**
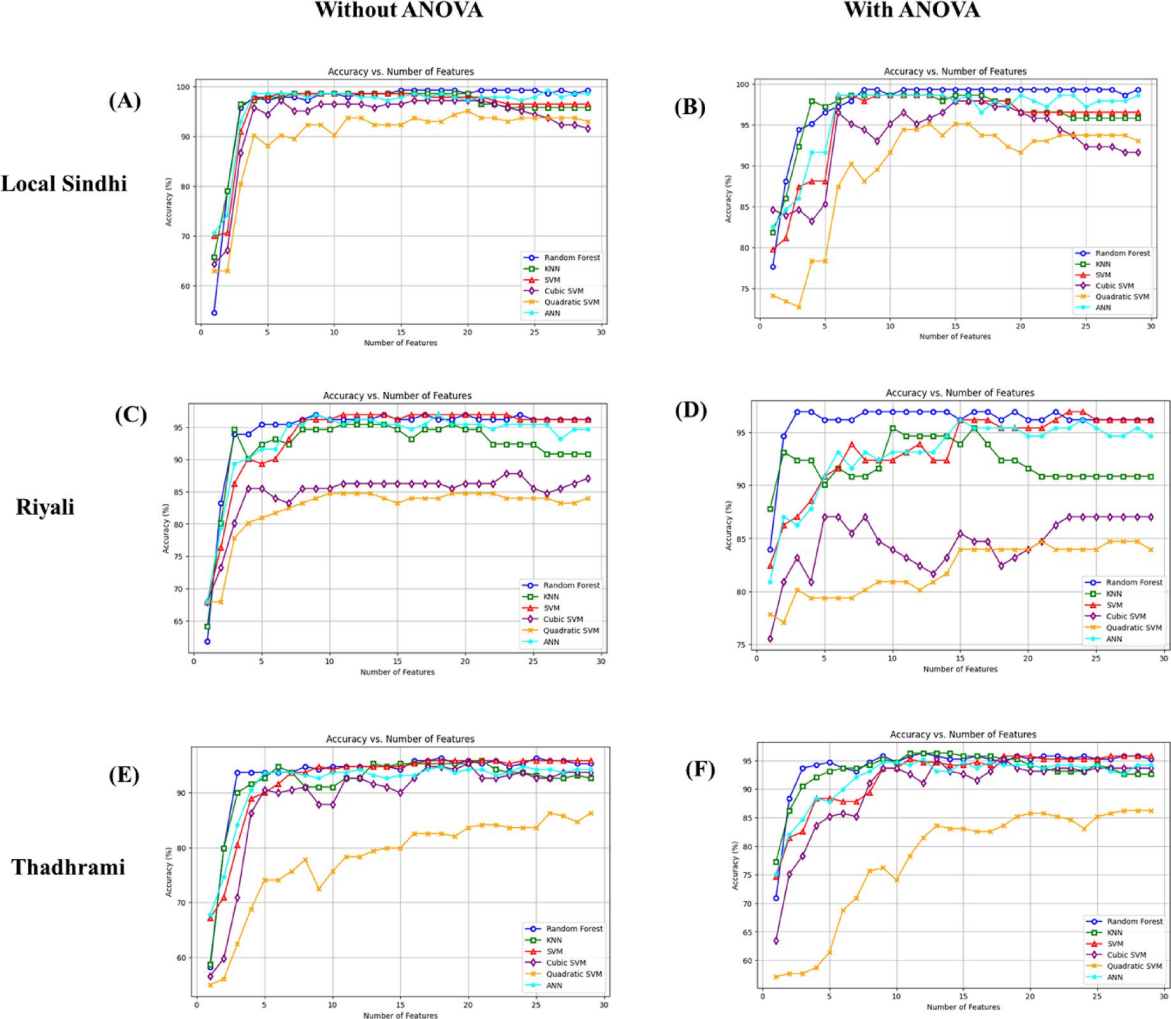
Classification accuracy of different varieties of guava in different feature space dimensions obtained with and without feature selection for six different machine learning classifiers (**A**) local Sindhi without ANOVA (**B**) local Sindhi with ANOVA (**C**) Riyali without ANOVA (**D**) Riyali with ANOVA (**E**) Thadhrami without ANOVA (**F**) Thadhrami with ANOVA.

The classification accuracy of guava’s three varieties (Local Sindhi, Riyali, and Thadhrami) was determined using six different machine learning algorithms (RF, KNN, ANN, SVM, cubic SVM, and quadratic SVM). The guava image color, texture and shapes were used as different feature combinations to determine the classification accuracy, with and without ANOVA. The classification accuracy was varied with the number of features, as shown in Fig. 4. The importance of features is presented in supplementary figure S1, S2 and S3. Among the tested models, in all varieties, the RF and SVM classifiers have achieved the highest classification accuracy in different feature spaces for both with and without ANOVA. However, the quadratic and cubic SVM classifiers showed the lowest classification accuracy compared to the other classifiers due to the fixed quadratic boundary, which resulted in poor feature scaling.

Further, the model accuracy was used to determine the performance of each classifier. Because the accuracy was considered as the most important evaluation metric to assess the model performance. Additionally, model performance comparison was performed for each classifier for the development and designing of feasible, accurate and precise maturity classification model. In the Local Sindhi variety, the RF classifier showed the highest accuracy (99.3%) with and without ANOVA. In case of ANOVA, the K (number of features) = 8, for all three stages (green, mature green and ripe). The result showed no changes were found in the accuracy of the RF classifier with and without using ANOVA, in the Local Sindhi variety.

**Fig. 5:**
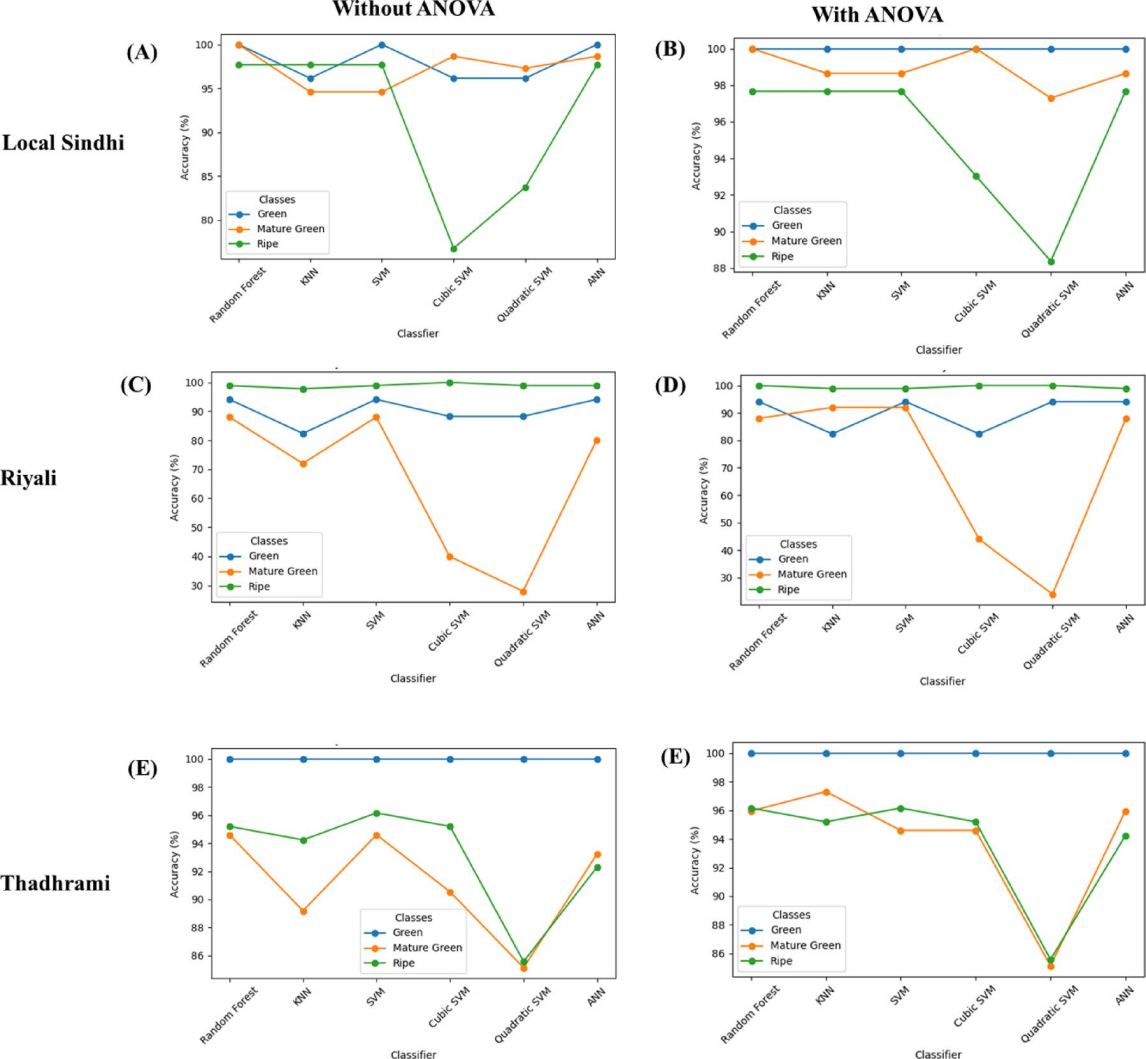
Model wise accuracy in each class (green, mature green and ripe) (**A**) without ANOVA local Sindhi (**B**) with ANOVA local Sindhi, (**C**) without ANOVA Riyali (**D**) with ANOVA Riyali, (**E**) without ANOVA Thadhrami (**F**) with ANOVA Thadhrami.

Further, in the case of the Riyali variety, the RF classifier showed the highest accuracy (96.2%) without using ANOVA, while the SVM classifier showed the highest accuracy (96.9%) with ANOVA, where K = 23 for all three stages. In, Thadhrami variety, the SVM algorithm showed the highest accuracy (95.8%) without ANOVA and RF classifier showed the highest accuracy (96.3%) with ANOVA, where K = 12 for all three stages. Similarly, no significant difference in the accuracy level was found with and without ANOVA in the Riyali and Thadhrami varieties using RF and SVM classifiers. The result suggested that the random forest was found more accurate classifier for guava fruit classification. The classification accuracy of models in each class is presented in Fig. 5.

In the present study, the classification model accuracy was found in the following order; (i) For Local Sindhi variety the accuracy order was; Random Forest > SVM = ANN = KNN > Cubic SVM > Quadratic SVM; and Random Forest > ANN > SVM > KNN > Cubic SVM > Quadratic SVM for both with and without ANOVA respectively. (ii) For Riyali variety with and without ANOVA, the order was; SVM > Random Forest > ANN > KNN > Cubic SVM > Quadratic SVM and Random Forest > SVM > ANN > KNN > Cubic SVM > Quadratic SVM, respectively. (iii) For Thadhrami variety, the order was found as, Random Forest > KNN > SVM > ANN > Cubic SVM > Quadratic SVM and SVM > Random Forest > Cubic SVM > ANN > KNN > Quadratic SVM with and without ANOVA, respectively. The overall accuracy of each classifier against each variety is presented in Table 1.

**Table 1:** Overall accuracy of each classifier with and without ANOVA.

S. no.	Algorithms	Local Sindhi (%)	Local Sindhi with ANOVA (%)	Riyali (%)	Riyali with ANOVA (%)	Thadhrami (%)	Thadhrami with ANOVA (%)
1	Random Forest	99.3	99.3	96.2	96.8	95.2	96.3
2	KNN	95.8	98.6	90.8	95.4	92.6	96.2
3	SVM	96.5	98.6	96.1	96.9	95.8	95.8
4	Cubic SVM	91.6	97.9	87	87.0	93.6	95.2
5	Quadratic SVM	93	95.1	83.9	84.7	86.2	86.2
6	ANN	98.6	98.6	94.6	96.1	93.1	95.2

**Fig. 6:**
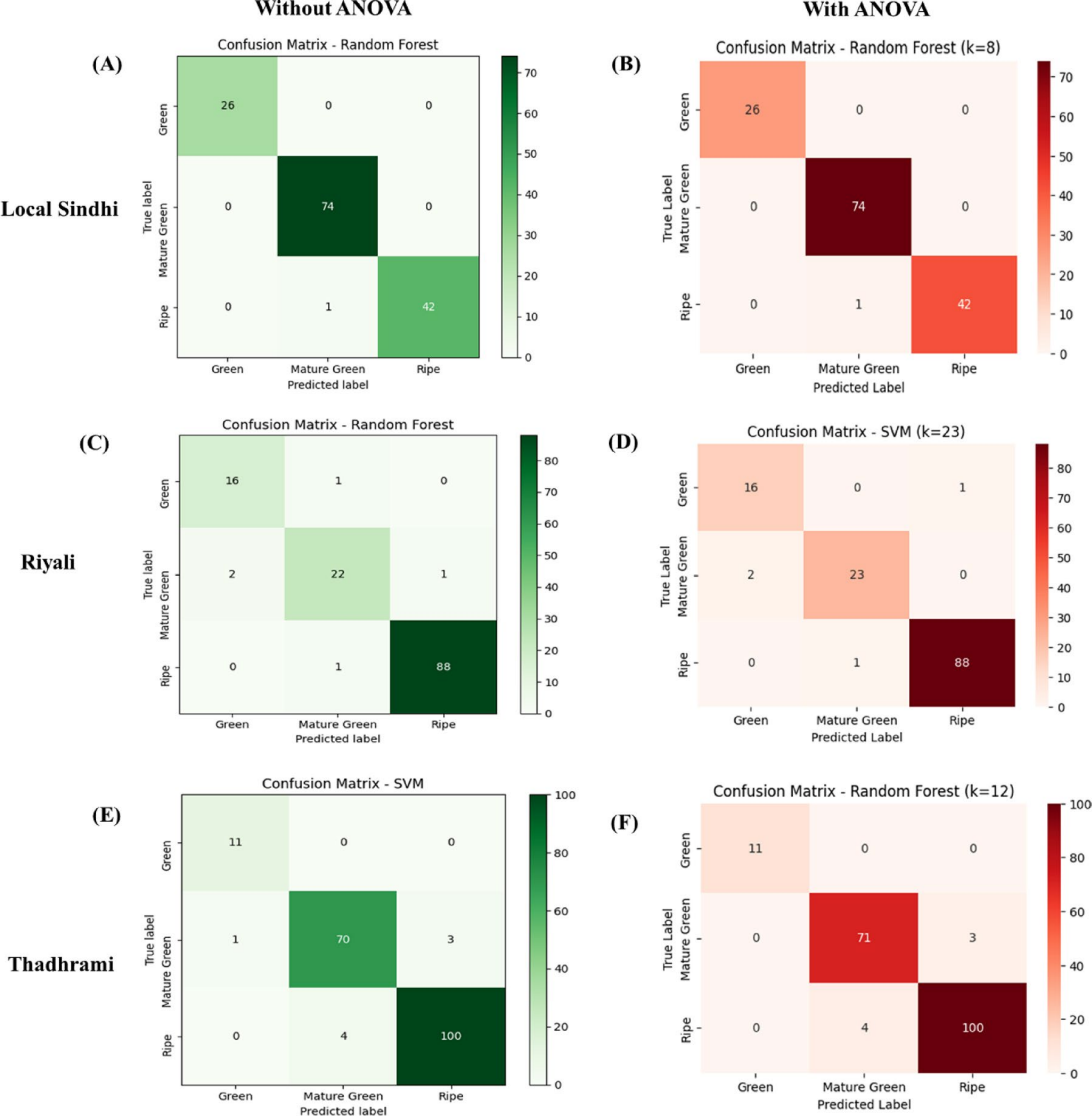
Confusion metrics of different varieties of guava (**A**) without Anova Local Sindhi (**B**) with Anova local Sindhi, K (number of features), (**C**) without Anova Riyali (**D**) with Anova Riyali, (**E**) without ANOVA Thadhrami (**F**) with ANOVA Thadhrami.

The classification model performance was further determined in terms of their precision, recall, F1 score and specificity. The confusion matrix was used to compare the actual and predicted stage identification between different training functions with/without ANOVA for the best represented classifier (Fig. 6). The best classifiers were found as; RF for Local Sindhi variety; SVM and RF for Riyali variety, and RF and SVM for Thadhrami variety for both with and without ANOVA. The results showed that the RF algorithm showed TP (Green) = 26, TP (Mature Green) = 74 and TP (Ripe) = 42; TN (Green) = 117, TN (Mature Green) = 68, TN (Ripe) = 100; FP (Green) = 0, FP (Mature Green) = 1, FP (Ripe) = 0; FN (Green) = 0, FN (Mature Green) = 0, and FN (Ripe) = 1 for Local Sindhi with and without ANOVA respectively. In the Riyali variety, the SVM and RF showed the highest accuracy as discussed above. Therefore, TP, TN, FP, and FN values are presented for both SVM and RF. The result obtained from confusion matrix for Riyali variety using RF algorithm showed; TP (Green) = 16, TP (Mature Green) = 22 and TP (Ripe) = 88; TN (Green) = 112, TN (Mature Green) = 104, TN (Ripe) = 41; FP (Green) = 2; FP (Mature Green) = 2, FP (Ripe) = 1; FN (Green) = 1, FN (Mature Green) = 3, and FN (Ripe) = 1, without using ANOVA. The result obtained from confusion matrix for the SVM algorithm showed TP (Green) = 16, TP (Mature Green) = 23 and TP (Ripe) = 88; TN (Green) = 112, TN (Mature Green) = 105, TN(Ripe) = 41, FP (Green) = 2, FP (Mature Green) = 1, FP (Ripe) = 1, FN (Green) = 0, FN (Mature Green) = 2, and FN (Ripe) = 1 for Riyali variety with ANOVA.

**Fig. 7:**
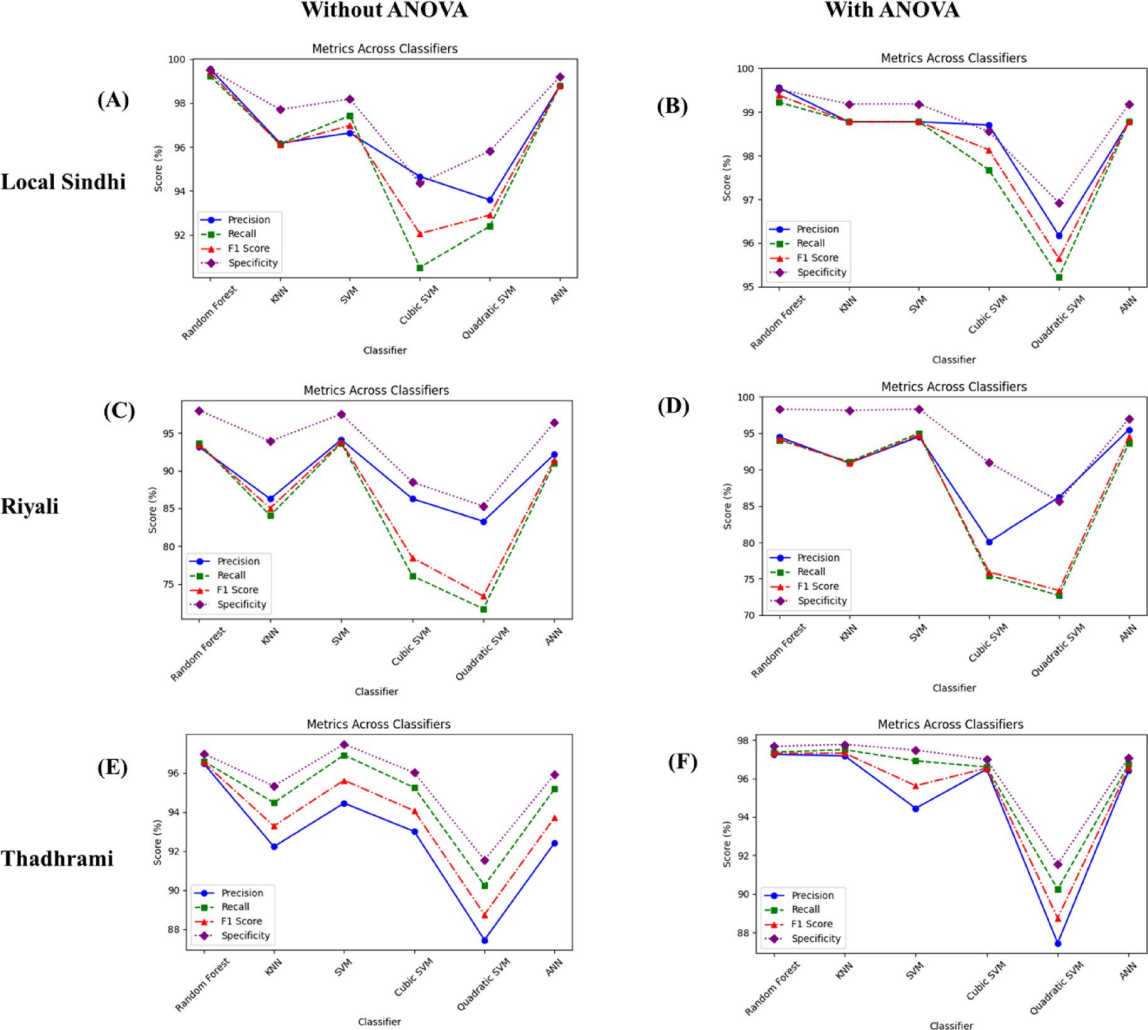
Metrics score verse classifiers (**A**) without ANOVA local Sindhi (**B**) with ANOVA local Sindhi (**C**) without ANOVA Riyali (**D**) with ANOVA Riyali (**E**) without ANOVA Thadhrami, and (**F**) with ANOVA Thadhrami.

Similar to the Riyali variety, in the case of Thadhrami, the RF and SVM showed the highest accuracy. Therefore, TP, TN, FP and FN values are presented for both SVM and RF. The result of confusion matrix for the RF algorithm showed TP (Green) = 11, TP (Mature Green) = 71 and TP (Ripe) = 100; TN (Green) = 178, TN (Mature Green) = 111 and TN (Ripe) = 82, FP (Green) = 0, FP (Mature Green) = 4, FP (Ripe) = 3, FN (Green) = 0, FN (Mature Green) = 3, and FN (Ripe) = 4 for Thadhrami variety with using ANOVA. The result of confusion matrix for the SVM algorithm showed TP (Green) = 11, TP (Mature Green) = 70 and TP (Ripe) = 100; TN (Green) = 177, TN (Mature Green) = 111 and TN (Ripe) = 82; FP (Green) = 1, FP (Mature Green) = 4, FP (Ripe) = 3; FN (Green) = 0, FN (Mature Green) = 4, and FN (Ripe) = 4 for Thadhrami variety without ANOVA. The Precision, Recall, Specificity and F1-score of the proposed method for all varieties (Local Sindhi, Riyali and Thadhrami) of guava in different classifiers are shown in Fig. 7. It can be seen that the proposed method showed the best classification performance for RF followed by SVM classifiers with higher values of Precision, Recall, Specificity and F1-score. In the RF classifier, the Precision, Recall, Specificity and F1-score were found as 99.56%, 99.22%, 99.38%, and 99.52% respectively for Local Sindhi with and without ANOVA. The SVM classifier showed the Precision, Recall, Specificity and F1-score as 94.53%, 95%, 94.73%, 98.31% respectively, and the RF showed the Precision, Recall, Specificity and F1-score as 93.14%, 93.66%, 93.37%, 97.99% respectively, for Riyali with and without ANOVA. Further, the RF classifier showed the Precision, Recall, Specificity and F1-score as 97.25%, 97.37%, 97.31%, 97.66% respectively; and the SVM shows the Precision, Recall, Specificity and F1-score of 94.45%, 96.92%, 95.62%, 97.48% respectively, for Thadhrami variety with and without ANOVA.

## Discussion

In the present study, the combination of features to fully represent the maturity levels of fruits cannot avoid the information redundancy between different feature combinations. Therefore, ANOVA was used to select the features to avoid redundant information in distinct characteristics. The classification accuracy of guava fruits improves with a rise in the dimension of the feature space with three different feature combinations (color, texture, and shape). Because high dimension feature space can acquire more discriminative and consistent information related to the characteristics of the guava based on the relevant feature^31^. In case feature space has enough classification information, the classification accuracy reaches its peak, after which it drops slightly for these three different feature combinations^29^. The results are in agreement with the previous study that the number of features affects the model accuracy^32,33^. The result showed that overall, the RF classifiers provide good accuracy for almost all the varieties. Because, the RF classifiers were often more interpretable, visualize the different decision trees and understand how individual features contribute to the final decision. The RF classifier can operate well with little feature engineering, since it handles both linear and non-linear relationships and can deal with categorical features adequately^34^. Therefore, random forest was found more accurate classifier for guava fruit classification. The previous study showed that the RF classifier was found as best classifier for the classification of the fruits^34–37^because the RF classifier do not require distribution assumptions for quality classification^37^. However, the reduced classification accuracy in the quadratic and cubic SVM classifier were might be associated with the fixed quadratic boundary and complex decision boundaries, which resulted in poor feature scaling and tight fitting of the training data, when the dataset is very large or heterogeneous. Thus, the overfitting of the large datasets is the major cause of the reduced accuracy in these classifiers. The results are in agreement with the previous study that the quadratic and cubic SVM were unable to perform better with the non-linear and noisy data due to fixed boundaries and data overfitting^38^.

## Conclusion

The present study proposed an efficient, precise, and reproducible machine learning model for quality classification of different variety of guava. Among the six different machine learning classifiers (ANN, KNN, SVM, Cubic SVM, Quadratic SVM, and RF) used in the present study, the RF classifiers perform best for all three varieties of guava for their quality classification. The RF classifiers showed 99.3, 96.8 and 96.3% accuracy for the Local Sindhi, Riyali, and Thadhrami, respectively, with the feature selection using ANOVA. The slight variation in the accuracy level was attributed to the varietal difference of guava. The quadratic and cubic SVM classifiers showed the lowest classification accuracy due to the fixed quadratic boundary. Therefore, the result suggested that, RF classifier can be used to exploit the benefit of analyzing the quality characteristics using ANOVA, for different varieties of guava. Thus, the proposed model will help to automate the guava industries and reduce the postharvest losses and economic losses.

## Electronic supplementary material

Below is the link to the electronic supplementary material.

Supplementary Material 1.

## Data Availability

The detailed data can be retrieved from the Mendeley data repository using the provided link; https://data.mendeley.com/datasets/w3fg8jjmzr/1.

## Abbreviations

ML: Machine learning
ANN: Artificial neural network
KNN: k-Nearest neighbor
SVM: Support vector machine
RF: Random forest
CNN: Convolution neural network
CLAHE: Contrast limited adaptive enhancement
HSV: Hue, saturation, value
FP: False positive
FN: False negative
TN: True negative
TP: True positive
ROI: Region of interest
ANOVA: Analysis of variance

## References

[1] Bylappa, Y. et al. Three decades of advances in extraction and analytical techniques for guava (*Psidium Guajava* L.): A review. *Results Chem* **10**, (2024).

[2] Khan, F. I. et al. A comprehensive review on guava: Nutritional profile, bioactive potential, and health-promoting properties of its pulp, peel, seeds, pomace and leaves. *Trends Food Sci. Technol.* **156**, 104822 (2025).

[3] Mahitha, K. K. M. B. K. Postharvest application of calcium salts on fruit quality of guava during storage. *Int. J. Adv. Res. Ideas Innov. Technol.* **4**, 381–385 (2018).

[4] Mehmood, A., Ahmad, M. & Ilyas, Q. M. On precision agriculture: enhanced automated fruit disease identification and classification using a new ensemble classification method. *Agric* **13**, (2023).

[5] Ismail, N. & Malik, O. A. Real-time visual inspection system for grading fruits using computer vision and deep learning techniques. *Inf. Process. Agric.* **9**, 1–14 (2021).

[6] Science, C. et al. Artificial intelligence for evalution of quality in fruits ripening. *Int. J. Nov Res. Dev.* **9**, 856–861 (2024).

[7] Mesa, A. R. Multi-Input deep learning model with RGB and hyperspectral imaging for banana grading. *Agriculture* 1–18 (2021).

[8] Kumari, A. & Singh, J. Fruits (Banana and Guava) datasets for non-destructive quality classifications. *Mendeley Data*. 10.17632/56td5w4wz2.1 (2024).

[9] Azarmdel, H., Jahanbakhshi, A., Mohtasebi, S. S. & Muñoz, A. R. Evaluation of image processing technique as an expert system in mulberry fruit grading based on ripeness level using artificial neural networks (ANNs) and support vector machine (SVM). *Postharvest Biol. Technol.* **166**, 111201 (2020).

[10] Sherafati, A., Mollazade, K., Koushesh Saba, M., Vesali, F. & TomatoScan An Android-based application for quality evaluation and ripening determination of tomato fruit. *Comput. Electron. Agric.* **200**, 107214 (2022).

[11] Apostolopoulos, I. D., Tzani, M. & Aznaouridis, S. I. A general machine learning model for assessing fruit quality using deep image features. *AI* **4**, 812–830 (2023).

[12] Abbas, H. M. T., Shakoor, U., Khan, M. J., Ahmed, M. & Khurshid, K. Automated sorting and grading of agricultural products based on image processing. *8th Int. Conf. Inf. Commun. Technol. ICICT 2019* 78–81 (2019). 10.1109/ICICT47744.2019.9001971.

[13] Hayat, A., Morgado-Dias, F., Choudhury, T., Singh, T. P. & Kotecha, K. FruitVision: A deep learning based automatic fruit grading system. *Open Agric.* **9**, (2024).

[14] Farisqi, B. A. & Prahara, A. Guava fruit detection and classification using mask Region-Based convolutional neural network. *Bul Ilm Sarj Tek Elektro* **4**, 186–193 (2022).

[15] Maitlo, A. K., Aziz, A. & Raza, H. A novel dataset of guava fruit for grading and classification. *Data Br.* **49**, 109462 (2023). 10.1016/j.dib.2023.109462 PMC10412757 37577735

[16] Murcia-Gómez, D., Rojas-Valenzuela, I. & Valenzuela, O. Impact of image preprocessing methods and deep learning models for classifying histopathological breast cancer images. *Appl Sci* **12**, (2022).

[17] Sahu, D. & Dewangan, C. Identification and classification of mango fruits using image processing. *Int. J. Sci. Res. Comput. Sci. Eng. Inf. Technol.* **2**, 203–210 (2017).

[18] Zhao, Z., Hicks, Y., Sun, X. & Luo, C. Peach ripeness classification based on a new one-stage instance segmentation model. *Comput. Electron. Agric.* **214**, 108369 (2023).

[19] Fu, L. et al. Banana detection based on color and texture features in the natural environment. *Comput. Electron. Agric.* **167**, 105057 (2019).

[20] Kang, J. & Gwak, J. Ensemble of multi-task deep convolutional neural networks using transfer learning for fruit freshness classification. *Multimed Tools Appl.* **81**, 22355–22377 (2022).

[21] Septiarini, A. et al. Machine vision for the maturity classification of oil palm fresh fruit bunches based on color and texture features. *Sci. Hortic. (Amsterdam)*. **286**, 110245 (2021).

[22] Eriksson, I. & Tabachnikova, N. Prediction of Total Soluble Solids and pH of Strawberry Fruits Using RGB, HSV and HSL Colour Spaces and Machine Learning Models. *Foods* **10**, (2022). 10.3390/foods11142086 PMC9318015 35885329

[23] Li, Y., Feng, X., Liu, Y. & Han, X. Apple quality identification and classification by image processing based on convolutional neural networks. *Sci. Rep.* **11**, 16618 (2021). 34404850 10.1038/s41598-021-96103-2 PMC8371106

[24] Kumari, S., Kumar, A. & Kumar, P. Maturity status classification of papaya fruits based on machine learning and transfer learning approach. *Inf. Process. Agric.* **8**, 244–250 (2021).

[25] Simon, P. & Uma, V. Deep learning based feature extraction for texture classification. *Procedia Comput. Sci.* **171**, 1680–1687 (2020).

[26] Nanni, L., Ghidoni, S. & Brahnam, S. Handcrafted vs. non-handcrafted features for computer vision classification. *Pattern Recognit.* **71**, 158–172 (2017).

[27] Tripathi, M. K. & Maktedar, D. D. A role of computer vision in fruits and vegetables among various horticulture products of agriculture fields: A survey. *Inf. Process. Agric.* **7**, 183–203 (2020).

[28] Akter, T. et al. A comprehensive review of external quality measurements of fruits and vegetables using nondestructive sensing technologies. *J. Agric. Food Res.* **15**, 101068 (2024).

[29] Chen, R. C., Dewi, C., Huang, S. W. & Caraka, R. E. Selecting critical features for data classification based on machine learning methods. *J. Big Data* **7**, (2020).

[30] Ropelewska, E. et al. Cultivar discrimination of stored apple seeds based on geometric features determined using image analysis. *J. Stored Prod. Res.* **92**, 101800 (2021).

[31] Iguyon, I. & Elisseeff, A. An introduction to variable and feature selection. *J. Mach. Learn. Res.* **3**, 1157–1182 (2003).

[32] Georganos, S. et al. Less is more: optimizing classification performance through feature selection in a very-high-resolution remote sensing object-based urban application. *GIScience Remote Sens.* **55**, 221–242 (2018).

[33] Wang, W., Zhu, A., Wei, H. & Yu, L. A novel method for vegetable and fruit classification based on using diffusion maps and machine learning. *Curr. Res. Food Sci.* **8**, 100737 (2024). 38681525 10.1016/j.crfs.2024.100737 PMC11046067

[34] Santos Pereira, L. F., Barbon, S., Valous, N. A. & Barbin, D. F. Predicting the ripening of papaya fruit with digital imaging and random forests. *Comput. Electron. Agric.* **145**, 76–82 (2018).

[35] Piedad, E., Larada, J. I., Pojas, G. J. & Ferrer, L. V. Postharvest classification of banana (*Musa acuminata*) using tier-based machine learning. *Postharvest Biol. Technol.* **145**, 93–100 (2018).

[36] Ma, L. et al. Prediction of banana maturity based on the sweetness and color values of different segments during ripening. *Curr. Res. Food Sci.* **5**, 1808–1817 (2022). 36254243 10.1016/j.crfs.2022.08.024 PMC9568694

[37] Sun, J., Li, S. & Yao, X. A novel method for multi-feature grading of mango using machine vision. *J. Comput.* **31**, 65–77 (2020).

[38] Breiman, L. & Random forests. *Mach. Learn.* **45**, 5–32 (2001).

