# Supplementary material for: Designing of guava quality classification model based on ANOVA and machine learning
Source: Sci Rep. 2025 Sep 30;15:33920. doi: 10.1038/s41598-025-09684-7 (PMC12484561; doi:10.1038/s41598-025-09684-7)
Supplement: Supplementary file 1 — Supplementary Material 1. [file 41598_2025_9684_MOESM1_ESM.docx]

**Designing of guava quality classification model based on ANOVA and Machine learning**

Abiban Kumari*, Jaswinder Singh

Department of Computer Science and Engineering

Guru Jambheshwar University of Science and Technology, Hisar -125001, Haryana, India.

*Corresponding author: Tel.: +91-9729486731

E-mail address: [abibanlangaya@gmail.com](mailto:abibanlangaya@gmail.com)

1. **Material and methods**

**Details of dataset used in the study**

Supplementary Table 1. Detailed data description

| Guava Varieties | Green | Mature Green | Ripe | Total |
| --- | --- | --- | --- | --- |
| Local Sindhi | 98 | 360 | 253 | 711 |
| Riyali | 67 | 101 | 487 | 655 |
| Thadhrami | 67 | 368 | 508 | 943 |
| Total | 2309 | | | |

1. **Results**

Supplementary Figures:

Supplementary Figure S1: Feature importance of Local Sindhi across different classifiers.

Supplementary Figure S2: Feature importance of Riyali across different classifiers.

Supplementary Figure S3: Feature importance of Thadhrami across different classifiers.

**Data Availability Source**

1. A novel dataset of guava: <https://data.mendeley.com/datasets/w3fg8jjmzr/1>
